# Advancing development of medical countermeasures: Incorporating COVID-19 lessons learned into future pandemic preparedness planning

**DOI:** 10.1080/21645515.2022.2129930

**Published:** 2022-10-27

**Authors:** Robert A. Johnson, Richard C. White, Gary L. Disbrow

**Affiliations:** a Department of Health and Human Services, Biomedical Advanced Research and Development Authority, Administration for Strategic Preparedness and Response, Washington, DC, USA; b Booz Allen Hamilton, McLean, VA, USA

**Keywords:** Advanced research and development, BARDA, COVID-19, medical countermeasures, pandemic preparedness

## Abstract

The COVID-19 pandemic profoundly disrupted, and, out of necessity, accelerated innovation of research and development of medical countermeasures to combat COVID-19. Although countermeasures were developed with unprecedented speed as a result of decades of long-term Federal investments in platform technologies and existing partnerships, the pandemic also revealed gaps in our preparedness and response capabilities that threaten our readiness posture. Challenges include limited federal funding that hinders sustainable development and manufacturing of, and equitable access to, medical countermeasures. Here we discuss lessons learned from the development and production efforts of medical countermeasures, such as vaccines and immunotherapeutics, to combat COVID-19. This commentary highlights some of the key gaps and challenges that must be addressed to ensure preparation for future outbreaks caused by viruses of pandemic potential.

## Introduction

Since its inception in 2006 under the Pandemic All-Hazards Preparedness Act (PAHPA), the Biomedical Advanced Research and Development Authority’s (BARDA) mission has focused on the advanced development of medical countermeasures (MCMs) including vaccines, therapeutics, and diagnostic tools to address public health medical emergencies. Federal investments in the development of MCMs have provided licensed products that can be made readily available in response to outbreaks, as has been demonstrated during recent outbreaks of Ebola and monkeypox. MCMs also provide the capabilities to rapidly respond to outbreaks of new viruses, as was seen with the H1N1 influenza and COVID-19 pandemics.

The COVID-19 pandemic has validated previously understood challenges and identified new ones in the Nation’s pandemic preparedness and response capabilities. BARDA’s partnership model and portfolio approach, including investments in platform technologies, accelerated the development, regulatory authorization and approval, and delivery of critically needed MCMs in response to the COVID-19 pandemic. BARDA investments supported more than 130 COVID-19 partnerships leading to the regulatory approvals, authorizations, and/or procurement of 6 immunotherapeutics (i.e., 5 anti-spike protein monoclonal antibodies: bebtelovimab, Evusheld, bamlanivimab/etesevimab cocktail, REGEN-COV, sotrovimab; 1 anti-cytokine treatment: Actemra), 2 small molecule antiviral therapeutics (i.e., Lagevrio, Paxlovid), 4 vaccines (including mRNA, protein subunit, and vectored vaccines), and 29 diagnostics (including point-of-care and at-home tests).^1^ Further, more than 900 M doses of COVID-19 vaccines and 14 M treatment courses of therapeutics have been manufactured and delivered through these public-private partnerships. However, despite the accelerated speed with which these MCMs were developed and manufactured at scale, the time needed to make available necessary MCMs must improve if we are to meet the ambitious goals set forth in the White House’s American Pandemic Preparedness Plan (APPP). In short, a faster response to a future outbreak will require BARDA to make the right investments today. This article describes some of the lessons learned from BARDA’s COVID-19 MCM development and production efforts, and how BARDA is incorporating these lessons into its strategic plan^2^ consistent with the APPP.

## Lessons learned

### Medical countermeasures (MCMs), platforms, and rapid product development

BARDA’s comprehensive public-private partnership model and diverse MCM portfolio allowed a rapid pivot to, and accelerated development of, lifesaving MCMs to fight the COVID-19 pandemic. Key to the success of addressing the pandemic was the development and emergency use authorization (EUA) of a suite of MCMs, beginning with diagnostics and small molecule antiviral therapeutics, followed by immunotherapeutics and vaccines. Such a suite of MCMs enabled the U.S. Government to detect, treat, and prevent SARS-CoV-2 infection. The diversity of available MCMs and the platforms with which they were produced proved especially valuable as new SARS-CoV-2 strains evolved and rendered some MCMs less effective or even ineffective^3^—for example, as some monoclonal antibodies lost effectiveness against new variants, oral antivirals became available that helped fill the treatment gap for high-risk patients. One important lesson learned is:

- An effective response requires multiple therapeutics, diagnostics, and vaccines working in tandem to treat, diagnose, and prevent disease.

In February 2020, BARDA was able to pivot ongoing, multi-year partnerships with Moderna (vaccines) and Regeneron (monoclonal antibody cocktail), among others, to develop MCMs to address COVID-19. Partnerships included BARDA support for clinical and non-clinical development, and manufacturing scale-up and validation, and both programs achieved EUA less than 12 months from recognition of a potential pandemic – to put this accomplishment into perspective, until COVID-19, the fastest vaccine ever developed took 4 years from viral sampling to approval.^4^ Successful development of COVID-19 MCMs required significant U.S. Government investment in multiple MCM candidates with different underlying technologies – this reduced risk of one or more candidates failing to demonstrate safety or effectiveness at any point in the development process. Finally, as variants of the prototype SARS-CoV-2 virus arose, multiple countermeasures lost potency – demonstrating platforms and approaches are needed that provide better breadth and/or duration of protection. In summary, three important lessons learned from these efforts are:

- Having in place flexible MCM development and support contracts that can rapidly pivot to and evolve with new viral or bacterial pathogens are required.
- Platform technologies can accelerate MCM development, to the extent new pathogens can be “plugged” into existing platforms.
- Improved platform technologies that can be used to develop MCMs faster and with better breadth and duration of protection against new emerging infectious diseases are needed.

Rapid development of MCMs during the pandemic presented numerous operational challenges – including staffing, funding, and adjusting to the pandemic as it evolved. Lack of funding delayed start-up of some MCM development efforts. As funding became available, chokepoints around contracting efforts became apparent. This was partially addressed through the establishment of a strong partnership between BARDA and the Department of Defense (DoD), which provided extensive acquisition and operational support. In short:

- Funding at the start of a response is essential.
- Rapid operational scale-up capabilities require appropriate staffing and acquisition mechanisms be in place prior to an emergency to reduce response timelines.

Clinical development of COVID-19 therapeutics and vaccines also posed several challenges. BARDA supported different types of clinical trial operational models to test new and repurposed MCMs, including centrally managed platform trials, trials using protocols harmonized across developers, and trials with a single developer testing multiple products. The most successful of these models entailed partnering directly with MCM developers, utilizing their capabilities and resources to move their product rapidly through clinical development. By utilizing our partners’ flexible, adaptable infrastructures the trials could easily accommodate operational changes. Further, protocol endpoints and statistical power were reviewed by the U.S. Food and Drug Administration (FDA) under standard regulatory processes, ensuring that once successfully completed (i.e., the primary endpoint was met), clinical trial results would support a label claim. These approaches avoided challenges seen with many other protocols, enabling full, rapid protocol enrollment, and rapid regulatory approval – exemplified by Regeneron’s Phase 3 trial for its REGEN-COV monoclonal antibody cocktail, which was initiated in early June 2020, with results available by September, leading to an EUA in November. Finally, the Operation Warp Speed (OWS)/Countermeasures Acceleration Group effort enabled the acceleration of vaccine development by providing a whole-of-Government effort – bringing together numerous individual Federal components and expertise under one roof, with rapid, streamlined negotiating, budget/spending, and decision-making capabilities. This was perhaps best exemplified by the six large Phase 3 clinical efficacy trials for vaccine candidates. While companies had regulatory oversight and executed protocols under BARDA/DoD Joint Program Executive Office (JPEO) product development contracts, OWS established an overall framework that applied across trials, including alignment of key protocol attributes (e.g., primary endpoints, assays for correlates of protection).^5,6^ OWS also fostered collaborative protocol development, execution, and oversight between developers and experts from DoD and the Department of Health and Human Services, including the National Institute of Allergy and Infectious Diseases (NIAID) and BARDA, as well as individuals from the NIAID-funded COVID-19 Prevention Network (CoVPN).^7^ This approach allowed for general comparison amongst vaccines/trial results, while at the same time, allowing each vaccine to advance as quickly as possible and adjust protocol criteria to suit unique aspects of the product and/or development pathway. Lessons learned from this rapid response effort include:

- Partnering with developers to take individual MCMs from development through FDA licensure and manufacturing (end-to-end) enables operational efficiencies that reduce development time and cost.
- When developing multiple products for a similar indication, standardization of key attributes across protocols is beneficial.
- Leveraging MCM development partners’ existing infrastructure and capabilities as a force multiplier maximizes flexibility and efficiency.
- Working with FDA and industry to design pivotal clinical trials with sufficient power and robust endpoints is critical for expediting regulatory approval.

Finally, this success may not be possible to replicate with other existing or novel viruses – witness the decades and billions of dollars invested in development of other vaccines for viral threats such as hepatitis C virus, human cytomegalovirus, and human immunodeficiency virus (HIV). These challenges underscore the importance of continued research to identify new platforms and novel approaches to antigen design/composition. Furthermore, while COVID-19 vaccine development was incredibly fast, response times are fastest when already-licensed products are available and stockpiled – reducing response time from months to hours. For example, past BARDA investments resulted in licensed Ebola vaccines, therapeutics, and diagnostics available to ship immediately following identification of a recent outbreak.^8^ In conclusion:

- The best way to reduce risk and enable the fastest response is investing in development, licensure, and stockpiling of products against known pathogens prior to an outbreak.

### Manufacturing

During the pandemic, rapid development also presented numerous well-documented manufacturing challenges – specifically the limited availability of raw materials and supplies, equipment, trained workforce, and manufacturing capacity and capability impacted production of COVID-19 and non-COVID-19 MCMs.^5,9–11^ While significant investments in capacity expansion were made to address these limitations, with the decline in demand, it is increasingly challenging to maintain this capacity due to lack of available Federal funding to sustain them. Three key lessons learned from this effort are:

- Greater supply chains and manufacturing flexibility and capacity are required – this includes both upfront and sustained, long-term investments.
- Being capable of rapidly manufacturing MCMs domestically for pandemic response requires facilities to actively produce products between responses – having facility space and equipment alone (i.e., capacity), without trained staff, ongoing FDA-inspected processes, and active quality systems available is insufficient.
- Sustainable models to support the full duration of pandemic response capabilities, supply chains, and facilities interpandemic must be identified; without these, meeting the APPP timelines is not achievable.

### Ease of use and access

Responding to an emerging threat requires both development and uptake of MCMs. This can be impacted in two significant ways: (1) regulatory approval to use the product in as broad an age range as possible; and (2) product formulations/presentations that enable wide distribution, do not require specialized expertise to administer, and maximize convenience to the end user. As was seen throughout the COVID-19 response, product improvements that increased ease of use and broadened population access increased utilization.^12^ For example, formulation changes that allowed monoclonal antibodies to be delivered intramuscularly rather than intravenously dramatically increased the number of sites that could administer the product, directly increasing the number of patients that could be administered treatment.^13^ In contrast, as more vaccine booster doses were recommended, compliance with the recommendation dropped.^14^ Key lessons learned from this aspect of development are:

- Attributes that support widespread distribution and administration, minimize wastage, and maximize compliance with dosing requirements must be incorporated into MCM development.
- MCM development programs must include a plan to quickly cover as much of the US age range, as appropriate.
- Easy-to-use diagnostics that can be utilized in homes and other non-traditional testing settings greatly improve access to testing and have potential to reduce disease spread.

## Looking to BARDA’s future

**Figure 1.**
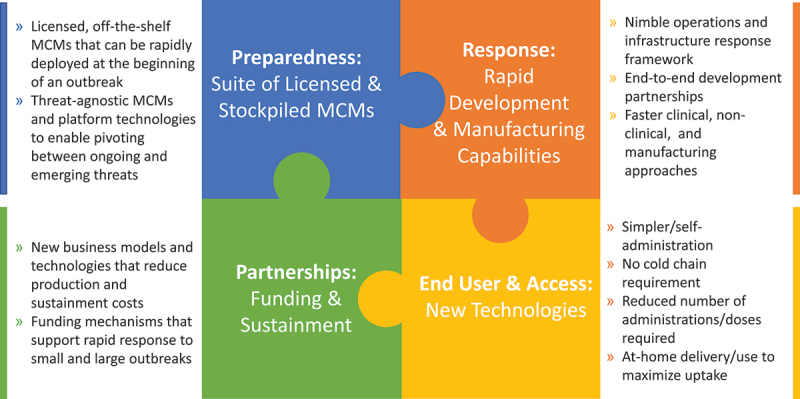
BARDA’s cross-cutting approach to pandemic preparedness and response.

The APPP outlines a whole-of-Government approach to preparing for and responding to the next viral pandemic. Based on several responses over the last 15 years, including H1N1, Zika, Ebola, and COVID-19, BARDA has identified four interconnected components (Figure 1) that provide a foundation for future MCM pandemic preparedness and response: a suite of available and stockpiled MCMs; rapid development and manufacturing capabilities; incorporation of new technologies targeting improved end-user experience; and funding and sustainment.

### Preparedness: suite of licensed and stockpiled MCMs

The most effective pandemic response requires us to have licensed and stockpiled diagnostics, therapeutics, and vaccines that can be immediately deployed as a threat emerges. While we cannot always predict the next threat, we can focus on some of the 26 viral families with pandemic potential,^15,16^ many of which do not yet have licensed vaccines or therapeutics. BARDA is committed to supporting the development, licensure, and availability of a suite of rapidly deployable MCMs to support responses to future viral outbreaks of known and novel pathogens.

Broad-spectrum oral antivirals and next-generation sequencing diagnostics that track and differentiate virus strains in real time will be of particular importance in preparing for the emergence of novel viruses. When looking at the ability to pivot existing tools and MCMs to a new threat, as demonstrated by the NIAID-led efforts with remdesivir, repurposing existing products is faster than development of new ones. Diagnostics that are better able to track and differentiate viruses will be key to identifying a new virus that emerges and supporting test-to-treat efforts.

Currently available platforms, particularly those for vaccines and monoclonal antibodies, do not yet have the characteristics necessary in terms of speed, breadth, or duration of protection to meet the ambitious APPP goals, nor can we be assured they will work for all pathogens. Significant investments in novel platform technologies will be critical to address these gaps.

### Response: rapid development and manufacturing capabilities

We do not know when or where the next outbreak will occur, whether MCMs will be available or must be developed, or the scale of the required response. Therefore, BARDA must establish a flexible, scalable response structure that can be implemented with short notice – but one that also can be sustained between pandemics. BARDA has already taken some steps to address these issues, e.g., putting in place more scalable support service contracts to address staffing gaps. New partnership and contracting approaches to more rapidly provide funding at the start of an outbreak are also in development. Four strategic approaches that were validated by the response and will remain core tenants are:

1. FDA approval of safe and effective MCMs to maximize confidence and uptake of the product is the ultimate objective.
2. Partnering with developers for an end-to-end MCM solution – including tapping into their capabilities, infrastructure, and resources as force multipliers – to accelerate product development.
3. Maintaining a nimble, flexible, and adaptable response framework that can rapidly pivot to address new threats.
4. Embracing new technologies and working with developers and regulators to integrate them into products and take them to regulatory approval.

Finally, current platforms and product development pathways are insufficient to achieve the APPP goal of developing a vaccine within 100 days after a pandemic threat appears, and producing enough vaccine for the U.S. population by 130 days and the world by 200 days.^17^ To achieve this, we must develop new platforms while also transforming the standard nonclinical, clinical, and manufacturing product development approaches – and do so in a way that is scalable, sustainable, and cost effective. Achieving even greater speed will require: (1) having candidate MCMs further along the development pipeline (e.g., no betacoronavirus vaccine candidate had advanced beyond Phase 1/2a trials prior to the COVID-19 pandemic);^18^ and (2) having existing infrastructure to support full-scale rapid manufacturing capacity to reduce the time between product authorization/licensure and distribution/administration. Additionally, innovative, adaptive trial designs and correlates of immune protection can shorten product development (e.g., streamlining administrative procedures to shorten enrollment, using surrogate endpoints that favor smaller sample size and/or shorter assessment period). Pandemic influenza serves as a case study where, because of the existence of an established correlate and manufacturing capability, H1N1 vaccine was developed, and distribution began less than 6 months after sequence identification.^19^ One could envision that an mRNA-based pandemic influenza vaccine, with the identified correlates of protection, could be produced in the timelines envisioned by the APPP.

### Ease of use and access: new technologies

In preparing for the next pandemic, new technology and innovation, such as on-demand manufacturing, will be critical to address current gaps in MCM development and manufacturing. Many of the advancements needed to address the lessons learned from COVID-19 MCMs regarding the end-user experience exist in early stages of development. These include formulations and devices that could ease storage requirements or make administration easier. Despite their promise, several challenges have delayed bringing these technologies to the mainstream, including: (1) uncertain regulatory pathways; (2) cGMP production capacity required; and (3) complexities in incorporating new technologies into an already approved MCM.

BARDA will continue to reduce this regulatory risk, through financial and technical partnerships such as the existing DRIVe Accelerator Network, BARDA Ventures, and its BLUE KNIGHT™ collaboration with Johnson & Johnson Innovation – JLABS. BARDA will also look to MCM development partners to integrate these attributes that will increase access at an earlier stage of product investment to reduce the amount of repeat and comparability studies needed. Finally, BARDA will continue fostering its numerous partnership opportunities for developers to advance innovative technologies that can be integrated into development and manufacturing practices for multiple MCMs and pandemic threats.

### Partnerships: funding and sustainment

BARDA supports products through the ‘valley of death,’ the activities between early development and regulatory licensure/commercial scale manufacturing. BARDA has a proven record in this space, supporting 64 FDA approvals, licensures, or clearances of products that cut across our threat space.^20^ This requires substantial time and funding. For context, the typical cost of advanced research and development (i.e., Phase 2 through launch), the space in which BARDA primarily operates, requires $11.50 for every $1 spent on early research and development (i.e., discovery through Phase 1).^21^ Further, the funding challenge remains after product approval is achieved – it shifts to maintaining regulatory approval, manufacturing capability, and, in many cases, stockpiling of the MCM so that it is available when needed. With each successful licensure, this sustainment funding requirement becomes larger. Adding to this challenge is the need to ensure affordable access to products during a response, such as in responding to Ebola and Monkeypox outbreaks.

Addressing these challenges will require an end-to-end management approach. Over the next several months BARDA will explore approaches to ensure that successes in MCM development and licensure can be sustained. As part of this effort, BARDA will seek innovative partnerships that address this issue in three ways:

- New business models and technologies that reduce per-dose production cost and cost of maintaining manufacturing capacity (without reducing readiness).
- Looking for collaborations and other approaches to achieve economies of scale across products.
- Developing public-private partnership models that both prepare for future pandemics as well as address current public health concerns.

This last point is of particular importance as it provides an opportunity to maximize the benefit of APPP investments by focusing on developing MCMs against viral strains that are both of pandemic potential and are currently circulating and causing human disease. This approach will not only improve pandemic preparedness, sustainability, and public health, but also potentially prevent emergence of new variants that are more infectious and could do greater harm domestically and globally.

## Conclusion

During the pandemic, BARDA delivered COVID-19 MCMs in record time, saving millions of lives and protecting the public’s health. As BARDA maintains its efforts in support of the COVID-19 response, we will build on these accomplishments and incorporate lessons learned to springboard to future successes preparing for and responding to the next pandemic. Thanks to decades of work, there are many MCM candidates currently in the pipeline that are in or are ready to enter advanced development to better prepare America against future outbreaks. BARDA’s ability to support these candidates to licensure and making them available will depend on adequate funding and identifying and supporting those candidates that address the gaps mentioned above.

Success will depend on selecting those candidates that incorporate lessons learned from COVID-19 development using new platform technologies, integration of new technologies earlier into the MCM development and regulatory process, achieving regulatory approval of products, and novel partnerships and business models that allow for streamlined operations and sustained manufacturing capabilities post-licensure. Of particular interest will be platform-based products that have a dual benefit – preparedness and response against future pandemics, as well as utilization against endemic diseases for which to date no or limited countermeasures exist – offering an immediate public health impact and preventing future pandemics before they start.
